# Long-Lasting Seborrheic Dermatitis Associated With Human Immunodeficiency Virus

**DOI:** 10.7759/cureus.91308

**Published:** 2025-08-30

**Authors:** Yoshihito Mima, Masako Yamamoto, Akira Nonogaki, Ken Iozumi

**Affiliations:** 1 Department of Dermatology, Tokyo Metropolitan Police Hospital, Tokyo, JPN; 2 Department of Dermatology, National Center for Global Health and Medicine, Tokyo, JPN

**Keywords:** cd4, human immunodeficiency virus, regulatory t cell, seborrheic dermatitis, t cell

## Abstract

Human immunodeficiency virus (HIV) infection is transmitted primarily through sexual contact, blood exposure, or from mother to child. The virus targets CD4-positive T cells, progressively impairing immune function and potentially advancing to acquired immunodeficiency syndrome (AIDS) if left untreated. HIV infection is known to be associated with various cutaneous and mucocutaneous conditions, including seborrheic dermatitis, psoriasis, oral candidiasis, herpes zoster, and Kaposi's sarcoma. We report a case of a 44-year-old man in whom HIV infection was early diagnosed after a treatment-refractory seborrheic dermatitis. He had a six-month history of persistent facial erythema, lichenification, and scaling that were unresponsive to topical therapy. Laboratory tests revealed HIV infection, with a markedly reduced CD4-positive T cells and elevated HIV RNA copy levels at diagnosis. After initiating antiretroviral therapy (ART), the immune status and the cutaneous symptoms significantly improved. Seborrheic dermatitis is frequently observed in patients with HIV infection, particularly in those with profound CD4 T cell depletion. This case highlights the potential for resistant seborrheic dermatitis to be an initial manifestation of HIV infection and underscores the importance of early diagnosis and therapeutic intervention.

## Introduction

Human immunodeficiency virus (HIV) infection is primarily transmitted through sexual contact, blood exposure (transfusions or shared needles), and vertical transmission from mother to child during childbirth or breastfeeding. HIV targets and gradually depletes CD4-positive T cells, leading to progressive immunosuppression. Without treatment, the disease may advance to acquired immunodeficiency syndrome (AIDS), characterized by opportunistic infections and malignancies [1,2].

HIV infection is known to be associated with a wide range of dermatologic and mucocutaneous conditions. Commonly reported manifestations include atopic dermatitis, psoriasis, pityriasis rubra pilaris, lichen planus, chronic eczema, oral candidiasis, molluscum contagiosum, oral hairy leukoplakia, herpes zoster, herpes simplex, seborrheic dermatitis, Kaposi's sarcoma, and eosinophilic folliculitis [3].

Herein, we report a case of a patient with seborrheic dermatitis revealing an HIV infection. Once the infection improved, the cutaneous symptoms of seborrheic dermatitis markedly resolved, suggesting a close association between the skin condition and the virus activity.

## Case presentation

**Figure 1:**
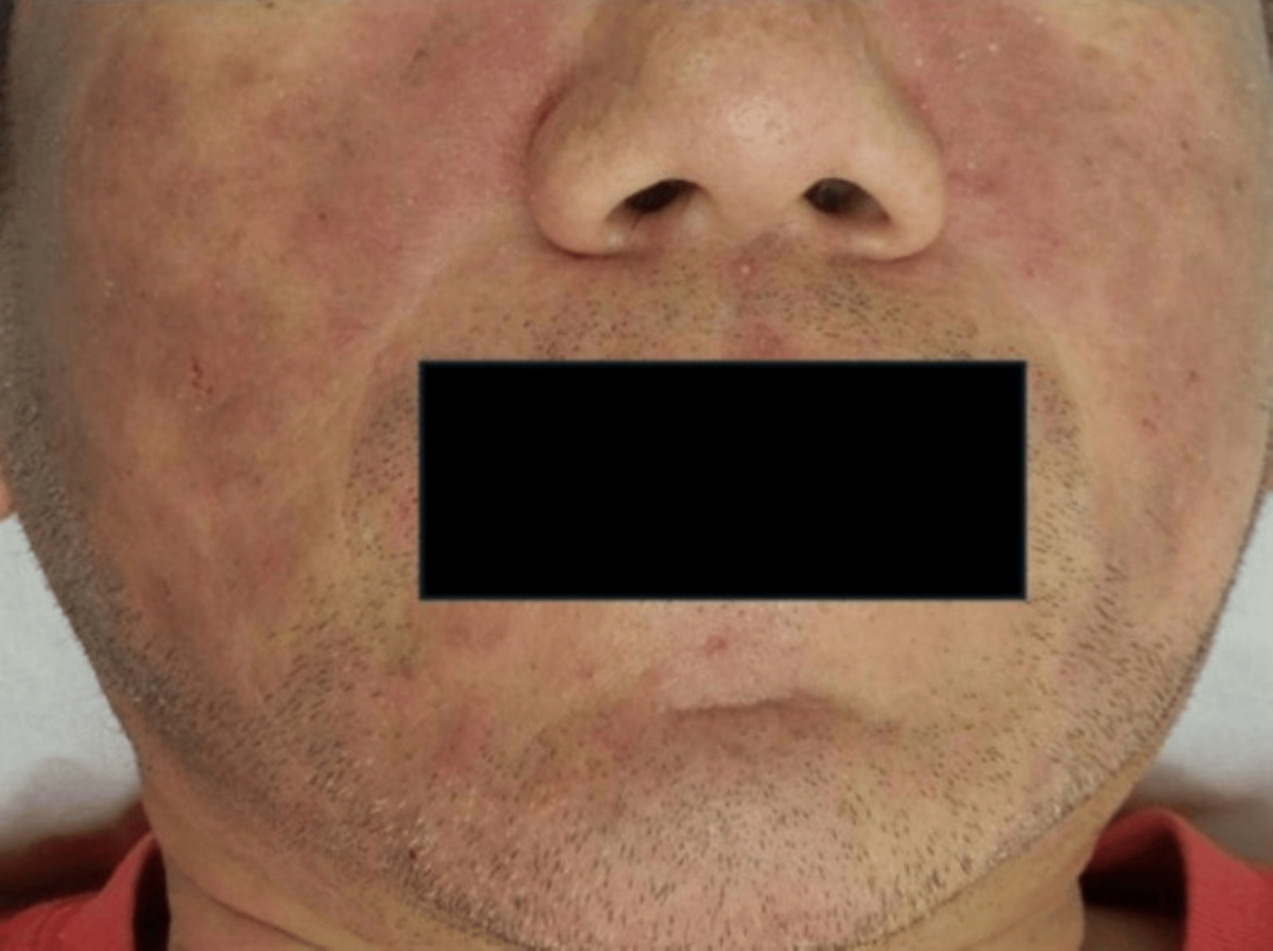
Clinical photograph: Pruritic facial erythema with lichenification and scaling, with post-inflammatory hyperpigmentation areas.

A 44-year-old man presented with a six-month history of persistent facial erythema accompanied by pruritus, lichenification, and scaling (Figure 1).

**Figure 2:**
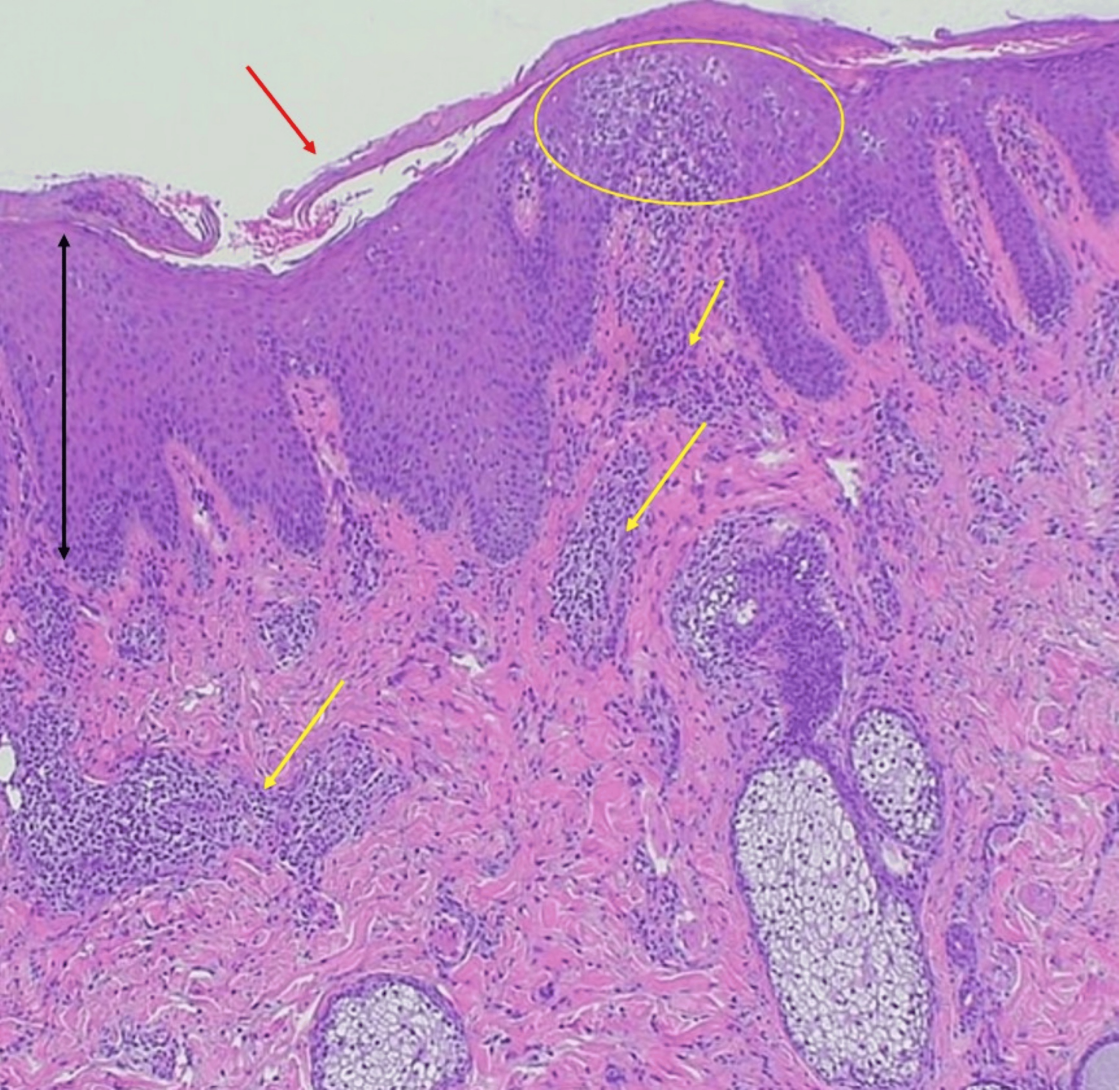
Histopathological finding: Histopathological examination revealed hyperkeratosis, parakeratosis (red arrow), and spongiosis (yellow circle) with epidermal thickening (black arrow) and prominent perivascular inflammatory cell infiltration in the dermis (yellow arrows). No neutrophilic infiltration within the stratum corneum or abnormal lymphocytes were observed (hematoxylin and eosin staining, x100).

Topical treatment with mild-class corticosteroids and ketoconazole had yielded insufficient improvement. The patient had a past history of syphilis, which had resolved five years earlier with prolonged amoxicillin therapy. He had no notable history of diabetes or other immune-related disorders, was not on any immunosuppressive medications, and had no prior episodes of photosensitivity. Histopathological examination of the facial lesion revealed hyperkeratosis, parakeratosis, acanthosis, and spongiosis. In the dermis, dilated capillaries and perivascular inflammatory infiltrates were observed (Figure 2).

However, Munro microabscesses or atypical lymphoid infiltrates were not detected. Fungal culture of the facial lesion was negative. Serological testing indicated past syphilis infection, while HIV testing revealed a markedly reduced CD4-positive T cell count of 27/μL and an HIV ribonucleic acid (RNA) load of 5.9 × 10⁶ copies/mL, confirming a diagnosis of HIV infection. All laboratory results are summarized in Table 1.

**Table 1:** The results of laboratory examination. RR = reference range; AST = aspartate aminotransferase; ALT = alanine aminotransferase; LDH = lactate dehydrogenase; CRP = C-reactive protein; IgG = immunoglobulin G; IgA = immunoglobulin A; IgM = immunoglobulin M; HIV = human immunodeficiency virus; T-SPOT = T-cell spot; Ab = antibody; RPR = rapid plasma reagin; TP = treponema; Hep = hepatitis.

Variable	Patient value	RR, adults
AST	66 U/L	11–33 U/L
ALT	89 U/L	6–37 U/L
Albumin	3.8 g/dL	3.8–5.0 g/dL
Total protein	7.7 g/dL	6.1–8.2 g/dL
Total bilirubin	0.8 mg/dL	0.2–1.2 mg/dL
LDH	258 U/L	135–214 U/L
CRP	0.2 mg/dL	<0.50 mg/dL
IgG	1740 mg/dL	870–1700 mg/dL
IgA	814 mg/dL	110–410 mg/dL
IgM	85 mg/dL	35–220 mg/dL
HIV 1/2 Ab, antigen	Reactive	Nonreactive
T-SPOT	Nonreactive	Nonreactive
RPR screen	Nonreactive	Nonreactive
TP antibody screen	Reactive	Nonreactive
Quantitative RPR level	0.6 R.U.	<1.0 R.U.
Quantitative TP antibody level	801 U/mL	<80 U/mL
Hep B Ab	Reactive	Nonreactive
Hep B DNA	Nonreactive	Nonreactive
Hep C Ab	Nonreactive	Nonreactive
CD4-positive T cell	27/μl	500-1600/μl
CD8-positive T cell	410/μl	150-1000/μl
HIV RNA	5.9×10⁶ copies	0 copies

The patient initiated antiretroviral therapy (ART) consisting of bictegravir sodium, emtricitabine, and tenofovir alafenamide fumarate. Three months later, CD4 T cell count increased to 343/μL, and the HIV RNA load decreased to 714 copies/mL. Concomitantly, the facial skin lesions responded well to topical prednisolone valerate and ketoconazole.

The absence of Munro microabscesses and restriction of lesions to the face made psoriasis unlikely. No evidence of atypical lymphoid proliferation was observed to suggest cutaneous lymphoma. The patient had no history of regular medications or photosensitivity before the onset, and after ART-induced remission, sun exposure did not trigger recurrence, making photosensitive dermatitis unlikely. The clinical content and the histopathological findings were consistent with an HIV-associated seborrheic dermatitis. The facial lesions have since resolved, dermatologic follow-up has been completed, and the patient has remained on ART.

## Discussion

In HIV infection, a wide range of cutaneous and mucocutaneous disorders, including seborrheic dermatitis, are known to be associated with immune dysregulation [3]. These HIV-related skin manifestations are thought to result, in part, from disruption in T-cell immunity due to a decline in CD4-positive T cells, including regulatory T cells [3]. Notably, seborrheic dermatitis has been significantly associated with marked reduction in CD4-positive T cell counts (<200/mm³), and is considered a potential clinical marker of advanced AIDS [4,5]. A Korean case series further demonstrated that conditions such as seborrheic dermatitis, irritant contact dermatitis, pruritus, and folliculitis occur significantly more frequently in HIV patients with CD4-positive T cell counts below 200×10⁶/L. Moreover, ART was shown to significantly reduce the incidence of seborrheic dermatitis and folliculitis in these patients [6]. In addition, HIV infection leads to a decrease in both CD4-positive T cells and regulatory T cells, with a relative increase in CD8-positive T cells, which may contribute to an elevated risk of drug hypersensitivity reactions [7]. These findings highlight that skin diseases in people with HIV are closely linked to abnormalities in T-cell-mediated immunity, particularly involving CD4 and CD8-positive T cells. Thus, dermatologic conditions such as seborrheic dermatitis are not merely superficial manifestations, but may serve as important clinical markers reflecting the underlying immune status of the patient [6,7].

With advances in ART, the mortality rate and incidence of opportunistic infections associated with HIV infection have significantly declined, and HIV is now regarded as a manageable chronic condition [1,2]. However, people with HIV remain at increased risk for cardiovascular diseases (CVDs), including myocarditis, cardiomyopathy, and atherosclerosis. These complications are thought to result from a combination of chronic inflammation, immune dysregulation, and metabolic abnormalities. Indeed, as ART has prolonged life expectancy, CVD has emerged as one of the leading causes of death in HIV-infected individuals [1,8]. Moreover, adverse effects of ART, such as dyslipidemia associated with protease inhibitors and cardiomyopathy linked to zidovudine-induced mitochondrial toxicity, have also been recognized as CVD risk factors [9]. These observations underscore the critical importance of early detection and timely initiation of treatment in HIV infection, which may not only improve overall prognosis but also reduce the risk of long-term complications, ultimately extending life expectancy [1,2,8,9].

In this case, early diagnosis of HIV infection was achieved by recognizing the clinical significance of treatment-resistant, chronic seborrheic dermatitis along with a past history of syphilis. Initiation of ART led to an increase in CD4-positive T cell count and a reduction in HIV RNA levels. These immunological improvements were accompanied by enhanced responsiveness of the skin lesions to topical treatment, resulting in marked clinical improvement. This course suggests a close association between the activity of HIV infection and the severity of seborrheic dermatitis in this patient. This case highlights the importance of considering underlying HIV infection when encountering atypical or treatment-refractory seborrheic dermatitis. A thorough review of the patient’s medical history is essential. Prompt diagnosis and early therapeutic intervention can help reduce the risk of HIV-associated chronic inflammatory conditions such as atherosclerosis and may contribute to improved long-term outcomes and life expectancy.

## Conclusions

HIV infection is known to be closely associated with various cutaneous conditions, including seborrheic dermatitis. Therefore, when encountering seborrheic dermatitis that is refractory to topical treatment, it is essential to take a thorough medical history and consider the possibility of underlying HIV infection. Early diagnosis and timely therapeutic intervention may help reduce the risk of HIV-associated chronic inflammatory conditions such as atherosclerosis, ultimately contributing to improved prognosis and prolonged survival.

## References

[1] Abarca YA, Chadalavada B, Ceron JR (2025). A comprehensive review of the manifestation of cardiovascular diseases in HIV patients. Cureus.

[2] Simon V, Ho DD, Abdool Karim Q (2006). HIV/AIDS epidemiology, pathogenesis, prevention, and treatment. Lancet.

[3] Bobotsis R, Brathwaite S, Eshtiaghi P, Rodriguez-Bolanos F, Doiron P (2024). HIV: inflammatory dermatoses. Clin Dermatol.

[4] Titou H, Ebongo C, Hjira N (2018). Dermatologic manifestations among human immunodeficiency virus patients in Morocco and association with immune status. Int J Dermatol.

[5] Ippolito F, Passi S, Di Carlo A (2000). Is seborrhoeic dermatitis a clinical marker of HIV disease?. Minerva Ginecol.

[6] Jung HJ, Ahn JY, Jang DH, Lee JI, Bae JY, Park MY (2019). Skin disease in Korean human immunodeficiency virus patient. Ann Dermatol.

[7] Yang C, Mosam A, Mankahla A, Dlova N, Saavedra A (2014). HIV infection predisposes skin to toxic epidermal necrolysis via depletion of skin-directed CD4⁺ T cells. J Am Acad Dermatol.

[8] Hmiel L, Zhang S, Obare LM (2024). Inflammatory and immune mechanisms for atherosclerotic cardiovascular disease in HIV. Int J Mol Sci.

[9] Henning RJ, Greene JN (2023). The epidemiology, mechanisms, diagnosis and treatment of cardiovascular disease in adult patients with HIV. Am J Cardiovasc Dis.

